# Advancements and challenges in inverse lithography technology: a review of artificial intelligence-based approaches

**DOI:** 10.1038/s41377-025-01923-w

**Published:** 2025-07-24

**Authors:** Yixin Yang, Kexuan Liu, Yunhui Gao, Chen Wang, Liangcai Cao

**Affiliations:** 1 Department of Precision Instruments, Tsinghua University, Beijing, 100084 China; 2 School of Materials Science and Engineering, Tsinghua University, Beijing, 100084 China

**Keywords:** Lithography, Applied optics

## Abstract

Inverse lithography technology (ILT) is a promising approach in computational lithography to address the challenges posed by shrinking semiconductor device dimensions. The ILT leverages optimization algorithms to generate mask patterns, outperforming traditional optical proximity correction methods. This review provides an overview of ILT’s principles, evolution, and applications, with an emphasis on integration with artificial intelligence (AI) techniques. The review tracks recent advancements of ILT in model improvement and algorithmic efficiency. Challenges such as extended computational runtimes and mask-writing complexities are summarized, with potential solutions discussed. Despite these challenges, AI-driven methods, such as convolutional neural networks, deep neural networks, generative adversarial networks, and model-driven deep learning methods, are transforming ILT. AI-based approaches offer promising pathways to overcome existing limitations and support the adoption in high-volume manufacturing. Future research directions are explored to exploit ILT’s potential and drive progress in the semiconductor industry.

## Introduction

In the realm of information technology, integrated circuits (ICs) are the core hardware components of modern devices^1–6^, including smartphones, computers, robotics, aerospace instruments, and the Internet of Things. Fabricated through advanced semiconductor processes, ICs drive technological innovation and shape daily life. According to Moore’s Law, transistor densities double approximately every 18 to 24 months^7^, leading to smaller feature size and higher integration. This trend highlights the critical role of lithography in IC fabrication. Lithography directly impacts the resolution and yield, driving the advancement of micro-scale and nano-scale fabrication technologies^8,9^. Techniques for fabricating binary optical elements and nano-structures range from electron-beam (EB) lithography^10–12^, femtosecond laser fabrication^13^, scanning-probe-based lithography^14,15^, direct laser writing^16–20^, two-photon lithography^21–25^, multi-photon lithography^26,27^, plasmonic nano-printing^28–30^, UV nanoimprint lithography^31^, ultrafast laser-induced nanostructuring^32^, photonic wire bonding^33^, phase-transition lithography^34^, laser materials processing^35,36^, multi-beam lithography^37^, to interference lithography^38^. Over time, lithography has evolved to meet higher transistor densities, improved performance, and reduced power consumption.

Lithography nodes are described by the critical dimension (CD), which determines the minimum feature size of a pattern^39^. As CDs shrink, maintaining high lithography resolution and pattern fidelity becomes challenging. According to the Rayleigh criterion^40^, resolution can be improved by reducing the exposure wavelength, increasing the numerical aperture (NA), and optimizing the process factor. The process factor is influenced by the illumination source, mask, and photoresist of the lithography system^41^. Initially, advancements focused on shorter wavelengths and higher NA, by using advanced exposure equipment. The earliest projection scanning lithography machine in 1973 had an NA of 0.167^42^. In 1990, Silicon Valley Group introduced a scanning lithography machine with a resolution of 0.5 μm. In 1995, Nikon released a 248 nm laser scanning lithography machine with a resolution of 0.25 μm. In 2004, Advanced Semiconductor Materials Lithography (ASML) introduced an immersion lithography machine with a wavelength of 193 nm, an equivalent NA of 1.35, and a resolution of 38 nm. In 2013, ASML launched an extreme ultraviolet (EUV) lithography machine with a wavelength of 13.5 nm, an NA of 0.33, and a resolution limit of 13 nm. The lithography wavelength evolved from g-line (436 nm), i-line (365 nm), KrF (248 nm), and ArF (193 nm) to 13.5 nm, which is adopted in industrial production. The NA has increased from 0.4 to 0.93 and then to 1.35. Over the decades, lithography has achieved remarkable milestones, from resolution of micrometers, to state-of-the-art EUV machines for features of 7 nm and beyond.

As feature sizes reach the nanometer scale, further reductions in exposure wavelength and increases in NA become increasingly complex and costly^39,41^. At this stage, resolution enhancement technologies (RETs) for reducing the process factor play a crucial role. Techniques such as optical proximity correction (OPC) and source mask joint optimization (SMO) fall under computational lithography^43^, which numerically models the lithography process and optimizes process variables. Computational lithography enables precise control and optimization of the fabrication process, improving resolution and pattern fidelity.

Computational lithography optimizes the illumination sources and masks according to the target wafer patterns. Among computational lithography techniques, the inverse lithography technique (ILT) stands out for mask pixelization and global optimization capabilities, generating near-optimal solutions of OPC. The ILT algorithm design needs to balance computational complexity, accuracy, and stability. Recent advancements in artificial intelligence (AI) have introduced transformative approaches to computational lithography and ILT^44–48^. Machine learning and deep learning techniques have shown promise in enhancing lithography modeling^49–71^ and improving algorithm efficiency^72–83^, marking a fertile ground for research.

By integrating AI with lithography modeling methods, researchers have achieved improvements in computational lithography accuracy and efficiency. For instance, machine learning improves thick mask simulations, three-dimensional (3D) effects, and near-field calculations^49–52^. Deep learning models such as convolutional neural networks (CNNs) and generative adversarial networks (GANs) reduce computational costs and improve pattern diversity and fidelity^53–57,59–64^. AI is also applied to etching processes, where neural networks and support vector machines (SVMs) optimize etch profiles and photoresist performance^67–70^. Hybrid methods combining genetic algorithms and physical modeling enhance the interpretability and robustness in photoresist development^65,66^. Model-driven deep learning improves prediction reliability by integrating prior physical models^81–84^. AI is a transformative force in computational lithography, driving progress toward higher precision, efficiency, and scalability in advanced manufacturing processes.

This work reviews recent advances in computational lithography, with a focus on emerging trends and challenges in ILT. We trace the evolution of computational lithography, from rule-based optical proximity correction (RBOPC), model-based optical proximity correction (MBOPC), to ILT. The historical development and theoretical framework of ILT are introduced. The application of AI in computational lithography and ILT is presented, including lithography modeling improvements and acceleration algorithms. Key challenges, such as optimization efficiency and mask manufacturing complexity, are discussed. We explore future perspectives and innovative design methodologies to advance ILT and support its adoption in high-volume IC manufacturing.

## The progress of computational lithography

**Fig. 1:**
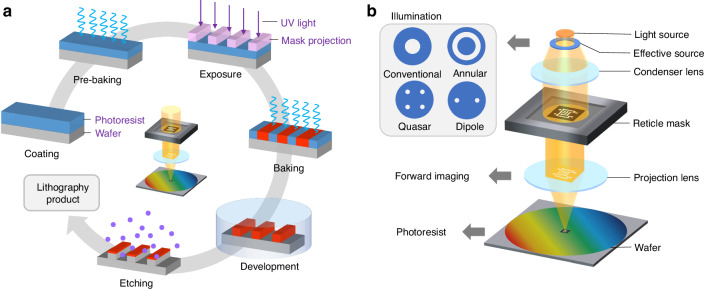
Schematic of the lithography process flow in IC manufacturing and the projection lithography system. **a** Principle of the lithography process in IC manufacturing. **b** Projection exposure system for photolithography

The common lithography process in IC manufacturing consists of several stages, including coating, pre-baking, exposure, baking, development, etching, resist stripping, and metrology^85^, as shown in Fig. 1a. The exposure step directly determines the quality of optical lithography. The optical lithography exposure has evolved through three main stages: contact, proximity, and projection. Early IC manufacturing relied on contact-proximity exposure, but contamination and mask damage were common issues. Due to the limited flatness of silicon wafers at the micron level, to avoid contact between the photoresist and the mask, proximity lithography also faced spatial resolution constraints^39^. To overcome the limitations, the first scanning projection lithography machine was introduced in 1973^42^. Since then, projection lithography has become the dominant technology for IC fabrication. In projection lithography, after coating with photoresist, a mask pattern is imaged onto the wafer. The mask pattern and imaging system determine which areas of the photoresist are removed, enabling pattern transfer, as shown in Fig. 1b. A key factor in lithography is the process window, which defines the widest possible range of process parameters. The process parameters include the exposure dose and focus. The process window reflects the robustness of the lithography process. A large process window indicates that the process can maintain stable output despite parameter fluctuations, while a narrow process window means high sensitivity to changes.

**Fig. 2:**
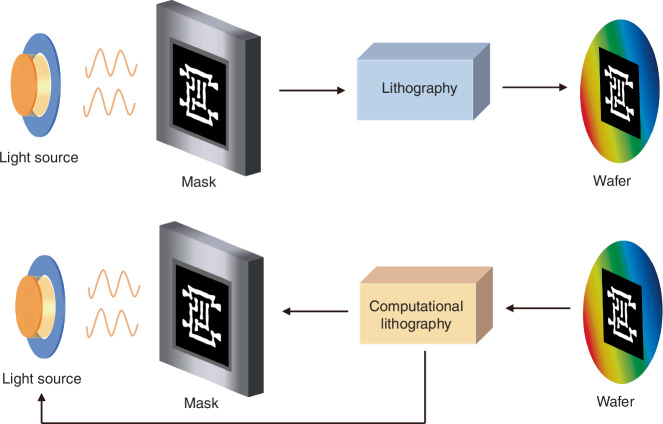
Forward imaging process of lithography and inverse process of computational lithography. In the forward imaging process, the optical system and mask determine the wafer patterns. In computational lithography, the optimal illumination source and mask are computed based on the desired target wafer pattern

RETs play a crucial role in improving lithography performance. Major RETs^86,87^ include off-axis illumination, OPC, phase shift mask, polarized illumination and SMO. In RETs, computational lithography models, simulates, and optimizes the lithography process^88^. The principle of computational lithography is shown in Fig. 2. The lithography model comprises two main components: the spatial imaging model, which describes the optical path from light source to wafer, and the photoresist model. The forward imaging process applies known optical parameters to predict wafer patterns. In contrast, computational lithography iteratively optimizes the illumination source and mask to achieve the best possible pattern^89^. The evolution of computational lithography has undergone several stages from inception to advancement: RBOPC^90^, MBOPC^91^, and ILT.

**Fig. 3:**
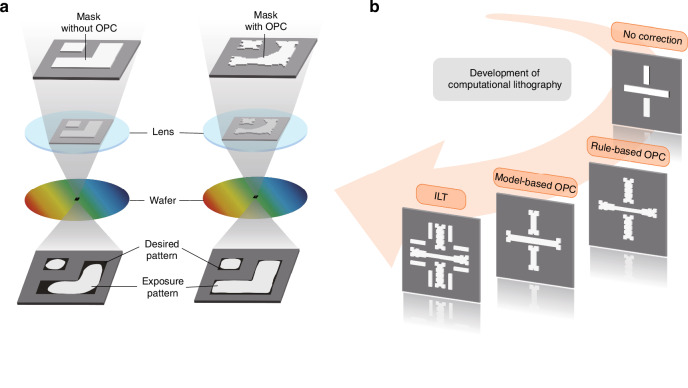
OPC in lithography and the evolution of computational lithography techniques. **a** OPE and OPC of optical lithography. The dark shape represents the desired pattern on the wafer, and the light shape represents the exposure pattern. Distortions of OPE include unequal line widths, line end shortening, and corner rounding. OPC makes pre-distortions on masks to reduce the distortions of exposure patterns, serving as a correction for OPE. The left side shows the exposure result of the mask without OPC, and the right side presents the exposure result of the mask with OPC. **b** The development of computational lithography includes RBOPC, MBOPC, and ILT

The optical proximity effect (OPE) describes that the image contrast and focus are affected by surrounding features^92^. Distortions of OPE include unequal line widths between isolated and dense areas, line end shortening, and corner rounding. The OPC mitigates the distortions by adjusting the mask pattern, as shown in Fig. 3a.

The development of computational lithography is shown in Fig. 3b. RBOPC^90^, the first generation of computational lithography, emerged from manual mask corrections and design rules. RBOPC relied on predefined correction rule tables, which stored adjustments for various pattern structures, such as different line widths, distances, line ends, convex and concave corners, and square holes. The precision depends on the details of the correction tables. During the correction process, this table is looked up according to the input pattern, and adjustments are made. Additionally, rule-based sub-resolution assist features (SRAFs) are also introduced to enhance the imaging quality of isolated lines. RBOPC was effective for sparse patterns at 250 nm and 180 nm nodes. However, RBOPC’s complexity increased exponentially with shrinking feature sizes, making rule table creation costly and impractical. As a result, MBOPC gradually replaced RBOPC.

MBOPC introduced a simulation-correction-feedback loop to refine the mask patterns^91,93^. MBOPC calculates the pattern by establishing models and optimizes the mask pattern contours. MBOPC consists of two approaches: edge-based optical proximity correction (EBOPC) and pixel-based optical proximity correction (PBOPC). EBOPC segments pattern edges into optimization variables and adjusts their positions to correct distortions. PBOPC pixelates the mask and optimizes the transmittance at each pixel to achieve the desired corrections. MBOPC was first implemented in 2001 for early 193 nm lithography systems^94^. SMO is another effective resolution enhancing approach^95,96^. SMO iteratively optimizes the illumination source and mask to achieve optimal imaging quality. At the 14 nm nodes, double patterning technology (DPT)^97,98^ further improved lithography resolution by dividing patterns into multiple exposures.

PBOPC later evolved into ILT, which computes the mask based on target pattern and lithography model^99^. ILT is characterized by mask pixelization and global optimization, making it capable of generating superior OPC solutions. Compared to its predecessors, ILT has the advantages of high imaging quality and large process window. Since it was proposed, ILT has received significant attention in academia and industry^100^. Nonetheless, its widespread adoption faces several challenges. ILT optimization is computationally intensive, limiting its use to local hot-spot correction rather than full-chip mask design. Curved masks generated by ILT are difficult to fabricate without simplification. With the advancement of AI, new approaches to computational lithography have emerged^44–48^. Additionally, multi-beam (MB) mask writing and deformation direct writing technologies have improved the manufacturability of curved masks^101^. The integration of AI-driven methods and optimized mask fabrication techniques presents promising avenues for computational lithography.

## Inverse lithography technology

### Forty years of inverse lithography technology

The proposal of ILT dates back to 1981, when B. E. A. Saleh et al. at the University of Wisconsin-Madison reported a pixel-based mask optimization method^99^. Starting with an initial guess, they flipped the mask pixels randomly, accepted the changes that improved the quality of the image pattern, and iterated until an optimal mask was achieved. In 1985, Nashold et al. developed an algorithm employing projection operators to optimize continuous-transmittance masks^102^. In 1991, Liu et al. utilized the branch-and-bound and simplex methods for mask optimization, introducing the “bacteria” algorithm to ensure the mask adhered to rule constraints^103^. In 2002, Rosenbluth et al. proposed an SMO algorithm to analyze the optimum diffraction spectrum, and compute ILT mask patterns^104^. Many other researchers contributed to the early foundation of ILT^105–107^.

Although early ILT methods theoretically improved lithography quality, most of them required huge computational costs. Limitations in optimization efficiency and mask manufacturability hindered the practical applicability. The emergence of immersion lithography^108^ alleviated the urgency of ILT research, allowing time for further exploration. Intel sponsored research in ILT, catalyzing a wave of research and initial commercialization.

In 2003, Luminescent Technologies applied ILT in semiconductor manufacturing for the first time, employing PSC algorithms for following fronts propagating with curvature-dependent speed^109^. In 2005, Luminescent announced the first ILT product, and Pang from Luminescent coined the term “inverse lithography technology”. ILT demonstrated superior lithography quality compared to OPC. In 2006, Abrams et al. optimized masks by addressing the rigorous inverse problem of lithography, improving depth of focus and exposure^110^. Hung et al. applied ILT to SMIC’s first 65 nm tape-out, achieving improved CD and a larger process window^111^. Lin et al. reported the differences and advantages of ILT over OPC^112^. ILT attracted the attention from companies including Graphics^113^, Intel^114,115^, and Startups^116^. In addition to companies, numerous universities contributed to ILT development. Between 2007 and 2008, Ma et al. developed generalized gradient-based RET optimization methods for ILT^117^, extending this framework to binary masks with coherent illumination^118^. The RET for gradient-based phase-shifting masks was also proposed^119^. Zhang et al. proposed a pixel-based mask representation for model-based ILT to enhance resolution and fidelity^120^. Shen et al. introduced a two-dimensional (2D) discrete cosine transform for ILT mask optimization^121^. In 2009, Yang et al. introduced a seamless-merging-oriented parallel ILT method^122^. Lam et al. demonstrated the application of computation lithography, using design-for-manufacturability and inverse lithography as examples^123^. In 2010, Jia et al. pioneered the integration of machine learning into ILT, treating mask design as a machine learning problem and incorporating focus variation as a stochastic variable to enhance robustness^124^.

By 2010, Luminescent, Intel, and Gauda had announced the utilization of ILT for full-chip design. Integration with illumination source optimization further improved performance. Despite continuous advancements over the decades, ILT remained primarily a hotspot correction technique rather than a full-chip mask solution. The challenge of excessive computational runtimes hindered the application of ILT in practical production.

Between 2013 and 2014, Lv et al. proposed a regularization framework based on mask filtering^125^, a conjugate gradient ILT algorithm^126^, and a cascadic multigrid method for robust inverse mask synthesis^127^. From 2009 to 2019, Shen et al. conducted a series of studies on level-set-based ILT. They solved ILT as an obstacle reconstruction problem, employing a level-set time-dependent model with finite difference schemes^128^. They considered defocus and aberration to enhance the robustness of layout patterns against process variations^129^, and introduced a vector imaging model^130^. A semi-implicit time discretization scheme for ILT was applied, enabling stable computation with large time steps^131^. In 2021, Shen et al. derived a generalized active contour model with a locally realized semi-implicit difference scheme, enhancing ILT optimization efficiency^132^. Yu et al. applied the Adam algorithm to mask optimization, leveraging momentum-based gradient adjustments to improve convergence speed and expand the process window^133^.

**Fig. 4:**
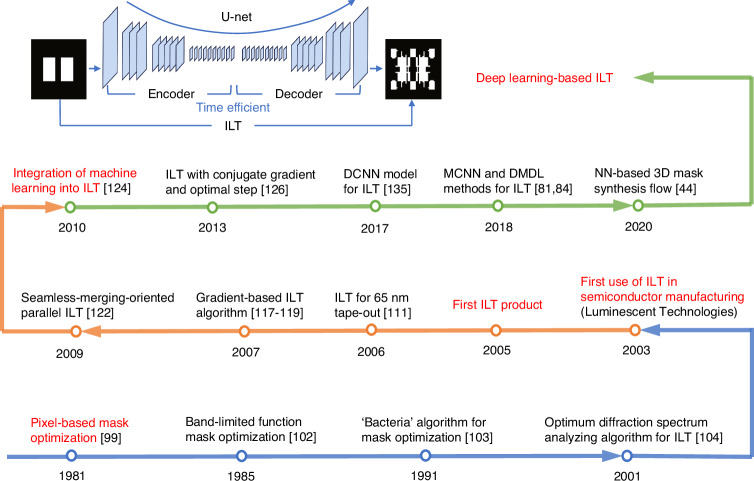
The development history and selected milestones of ILT

Although various gradient-based ILT algorithms were proposed, computational costs and efficiency remained significant challenges^134^. To address these issues, researchers turned to AI. In 2017, Wang et al. from ASML trained a deep convolutional neural network (DCNN) to assist full-chip SRAF placement^135^. Between 2018 and 2020, Ma et al. developed a model-driven convolutional neural network (MCNN) framework^81^, and later proposed a dual-channel model-driven deep learning (DMDL) method^84^, aiming to improve computational efficiency and imaging fidelity of ILT. In 2020, Liu from Synopsys demonstrated a network-based 3D mask synthesis flow, leveraging TensorFlow, reinforcement learning, and graphic processing unit (GPU) acceleration^44^. Deep learning is anticipated to propel GPU-based computing platforms into mainstream^100^. The forty-year developments of ILT are summarized in Fig. 4.

### Theoretical framework of inverse lithography technology

The lithography model serves as the foundation for computational lithography, encompassing two components: the spatial imaging model and the photoresist model. The Abbe theory describes the imaging process from the light source and mask to the wafer^136^. For the lithography system, partially coherent illumination (PCI) is employed. The extended light source is treated as multiple point light sources. Each point light source generates a coherent illumination light field on the image plane. Different point light sources remain incoherent, preventing interference between the light fields. Consequently, the spatial light intensity distribution is the sum of individual point sources and can be expressed as^43^

1 $$I({\hat{x}}_{i},{\hat{y}}_{i})={\int\int }_{\!-\infty }^{+\infty } S(\hat{f},\hat{g}){\left|{\int \int}_{\!-\infty }^{+\infty } H(\hat{f}+\hat{f}^{\prime},\hat{g}+\hat{g}^{\prime})B(\hat{f}^{\prime},\hat{g}^{\prime})\exp [-j2\pi ({\hat{x}}_{i}\hat{f}^{\prime}+{\hat{y}}_{i}\hat{g}^{\prime})d\hat{f}^{\prime}d\hat{g}^{\prime}]\right|}^{2}d\hat{f}d\hat{g}$$

where $I$ denotes the spatial image intensity, $S$ denotes the effective light source intensity distribution, $H$ denotes the transfer function of the projection system, and $B$ denotes the diffraction spectrum of the incident light after passing through the mask. $({\hat{x}}_{i},{\hat{y}}_{i})$ is the spatial coordinate, and$(\hat{f},\hat{g})$ is the frequency coordinate.

According to Hopkins frequency shift approximation, the diffraction spectrum of an off-axis light source point undergoes a frequency shift relative to an on-axis light source point. The Hopkins formula separates mask and optical system contributions. The imaging characteristic of the optical system is described by the transmission cross coefficient (TCC) as^43^

2 $$TCC(\hat{f}^{\prime},\hat{g}^{\prime};\hat{f}^{\prime\prime} ,\hat{g}^{\prime\prime} )={\int\int }_{\!-\infty }^{+\infty } S(\hat{f}^{\prime},\hat{g}^{\prime})H(\hat{f}+\hat{f}^{\prime},\hat{g}+\hat{g}^{\prime}){H}^{\ast }(\hat{f}+\hat{f}^{\prime\prime} ,\hat{g}+\hat{g}^{\prime\prime} )d\hat{f}d\hat{g}$$

The intensity distribution on image plane is

3 $$\begin{array}{l}I({\hat{x}}_{i},{\hat{y}}_{i})={\int\int\int\int}_{-\infty}^{+\infty}{TCC}({\hat{f}}^{\prime},{\hat{g}}^{\prime};{\hat{f}}^{\prime\prime},{\hat{g}}^{\prime\prime} )B({\hat{f}}^{\prime},{\hat{g}}^{\prime}){B}^{\ast}({\hat{f}}^{\prime\prime},{\hat{g}}^{\prime\prime} )\cdot \\ \exp [-j2\pi ({\hat{x}}_{i}({\hat{f}}^{\prime}-{\hat{f}}^{\prime\prime} )+{\hat{y}}_{i}({\hat{g}}^{\prime}-{\hat{g}}^{\prime\prime} ))]d{\hat{f}}^{\prime}d{\hat{g}}^{\prime}d{\hat{f}}^{\prime\prime} d{\hat{g}}^{\prime\prime} \end{array}$$

The TCC matrix is decomposed using singular value decomposition (SVD) based on its Hermitian symmetry as

4 $$TCC(\hat{f}^{\prime},\hat{g}^{\prime};\hat{f}^{\prime\prime} ,\hat{g}^{\prime\prime} )=\sum _{k}{\mu }_{k}{\phi }_{k}(\hat{f}^{\prime},\hat{g}^{\prime}){\phi }_{k}^{\ast }(\hat{f}^{\prime\prime} ,\hat{g}^{\prime\prime} )$$

where ${\mu }_{k}$ is the eigenvalue, and ${\phi }_{k}$ is the corresponding eigenvector. Based on the sum of coherent systems (SOCS) framework, the image intensity can be reconstructed as

5 $$I({\hat{x}}_{i},{\hat{y}}_{i})={\sum _{k}{\mu }_{k}|{F}^{-1}\{{\phi }_{k}(\hat{f}^{\prime},\hat{g}^{\prime})B(\hat{f}^{\prime},\hat{g}^{\prime})\}|}^{2}$$

where ${F}^{-1}\{\cdot \}$ is the Inverse Fourier Transform (IFT). For OPC and ILT, the optical system is the same while the mask pattern changes. The TCC is calculated only once.

The process of ILT includes the following steps. According to Hopkins theory, the optical system can be described by the TCC,

6 $$TCC({r}_{1};{r}_{2})=\sum _{k}\sum _{l}{p}_{k,l}{\varphi }_{k}({r}_{1}){\varphi }_{k}^{\ast }({r}_{2})$$

Then SVD is performed on TCC to obtain eigenvalues and analytical TCC kernels,

7 $$TCC({r}_{1};{r}_{2})=\sum _{i}{\mu }_{i}{h}_{i}({r}_{1}){h}_{i}^{\ast }({r}_{2})$$

According to the SOCS theory, the spatial image distribution can be represented by

8 $$I(r)={\sum _{i}{\mu }_{i}|{h}_{i}(r)\otimes M(r)|}^{2}$$

where $\otimes$ is the convolution, $M(r)$ is the mask. Photoresist models are generally described using sigmoid functions,

9 $$sig[I(r)]=\frac{1}{1+{e}^{-a(I(r)-tr)}}$$

where $a$ is the steepness, and $tr$ is the threshold of photoresist. The photolithography imaging is expressed by

10 $$Z(r)=sig[I(r)]=sig\left[{\sum _{i}{\mu }_{i}|{h}_{i}(r)\otimes M(r)|}^{2}\right]$$

Pattern error (PE) is described by L_2_ loss,

11 $$F\{M(r)\}={\left|\left| Z(r)-\tilde{Z}(r)\right|\right|}_{2}^{2}={\left|\left| sig[{\sum _{i}{\mu }_{i}|{h}_{i}(r)\otimes M(r)|}^{2}]-\tilde{Z}(r)\right|\right| }_{2}^{2}$$

where $\tilde{Z}(r)$ is the target pattern. The ILT problem for optimal mask $\tilde{M}(r)$ is expressed as

12 $$\tilde{M}(r)={\text{arg}}\,\min F\{M(r)\}$$

For inverse mask synthesis, researchers have been engaged in continuous expansion since pioneering work^137^. Based on the lithography model, ILT aims to compute a mask that generates the optimal wafer pattern^100^. ILT formulates a cost function to assess the wafer pattern quality. Various gradient-based optimization solvers are employed to optimize the cost function. A method illustrated here leverages the level set framework^109^. This approach is explored for production-level deployment, including Luminescent Technologies and Synopsys.

**Fig. 5:**
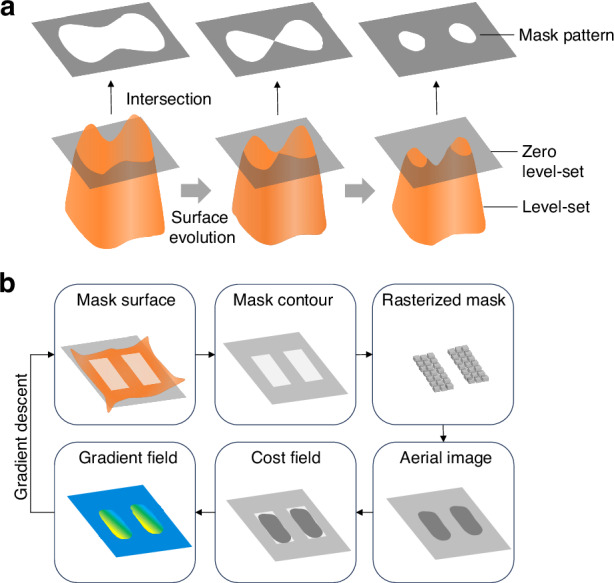
Mask optimization using level-set method and ILT workflow. **a** Level-set method for representing the mask optimization process. **b** The optimization procedure of ILT, including intersecting with the zero level-set for mask contour, rasterizing, computing the aerial image, calculating cost function and gradient, and optimizing gradient descent until getting the optimal mask pattern

The level set method tracks evolving interfaces by embedding 2D mask in a higher-dimensional surface. This converts mask evolution into a partial differential equation (PDE) problem. The intersection of the level set function with the zero-level set plane represents the mask contour, as shown in Fig. 5a. The mask is defined as

13 $$M(r)=\left\{\begin{array}{c}{m}_{\mathrm{int}},\phi (r) < 0\\ {m}_{{\rm{ext}}},\phi (r) > 0\end{array}\right.$$

and for binary masks, ${m}_{\mathrm{int}}=1$ and ${m}_{{\rm{ext}}}=0$. The distance function measures the shortest distance to the mask boundary,

14 $$d(r)=\,\min (|r-{r}_{\varOmega }|),{r}_{\varOmega }\in {M}_{\varOmega }$$

where ${M}_{\varOmega }$ is the mask contour. The level set function is defined as

15 $$\phi (r)=\left\{\begin{array}{l}-d(r),r\in {M}_{\varOmega -}\\ 0,r\in {M}_{\varOmega }\\ d(r),r\in {M}_{\varOmega +}\end{array}\right.$$

where ${M}_{\varOmega -}$ is the area outside the mask, and ${M}_{\varOmega +}$ is the area inside the mask.

The mask contour is embedded in a higher-dimensional surface, facilitating geometric topology evolutions. Then the problem is simplified as a PDE. The evolution of the level-set function is expressed as

16 $$\frac{\partial \phi }{\partial t}+{v}_{x}\frac{\partial \phi }{\partial x}+{v}_{y}\frac{\partial \phi }{\partial y}=0$$

where ${v}_{x}=\frac{dx}{dt}$ and ${v}_{y}=\frac{dy}{dt}$ denote the interface velocities along $x,y$ directions. The variation along the normal direction can be calculated from the Hamilton-Jacobi PDE,

17 $$\frac{\partial \phi }{\partial t}+v|\nabla \phi |=0$$

where $v=({v}_{x},{v}_{y})\cdot \nabla \phi /|\nabla \phi |$. Optimization proceeds by minimizing the cost function using gradient-based solvers. The optimization procedure of ILT is shown in Fig. 5b.

**Fig. 6:**
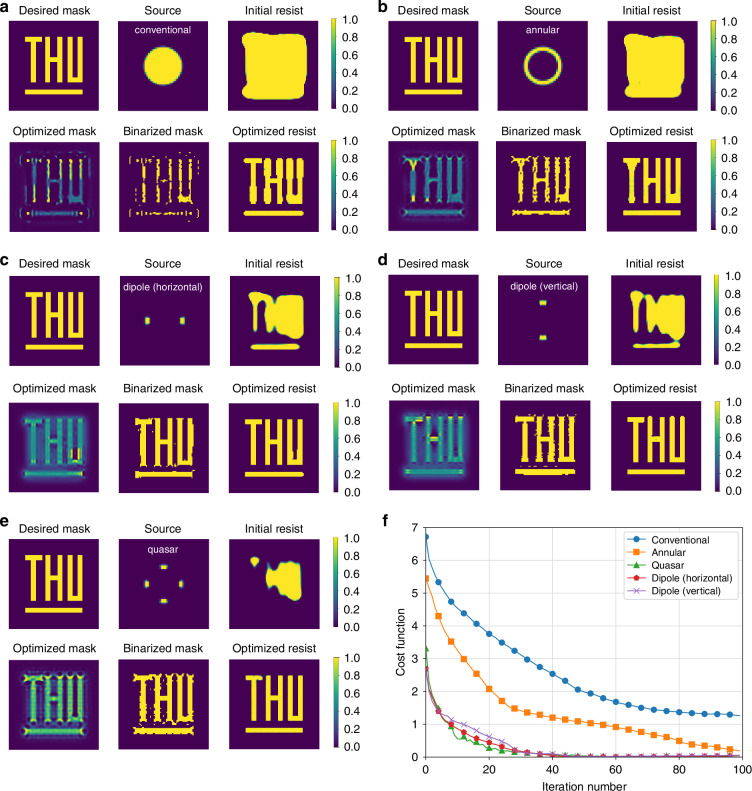
The ILT mask optimization simulation under different illumination sources. **a**–**e** show the illumination sources used in computational lithography, including conventional, annular, dipole (horizontal), dipole (vertical), and quasar. **a**–**e** show the initial resist patterns, the optimized masks, and the optimized resist patterns. **f** shows the convergence of the cost function with iteration number of **a**–**e**

In projection lithography, Köhler illumination ensures uniform mask illumination. The exit pupil of the illumination system overlaps with the entrance pupil of the projection objective, enabling PCI. Figure 6 presents numerical simulations of ILT mask optimization. Various illumination sources in computational lithography include conventional, annular, dipole, and quasar. These sources are tested in mask optimization simulations, as shown in Fig. 6a–e. A target pattern with the letters “THU” is designed as the input mask. Initial resist patterns on the wafer exhibit obvious OPE, causing distortions. Applying level set-based ILT with gradient descent yields an optimized mask and binarized version. The optimized resist pattern shows significant improvements in fidelity. Figure 6f depicts cost function convergence across different illumination conditions, demonstrating the efficiency of level set-based ILT.

## Artificial intelligence-based inverse lithography technology

The development of computational lithography primarily focuses on modeling and algorithms. Establishing lithography models requires balancing complexity and computational efficiency. Oversimplified models may lose critical physical details, leading to inaccurate optimization results. Complex models increase computational burdens, reducing algorithm efficiency. Algorithmic design needs to consider timeliness, accuracy, and stability. Timeliness ensures efficient computation, allowing rapid convergence. Accuracy ensures optimal and robust results. Stability reflects the algorithm’s ability to adapt to varying target structures.

Model-based computational lithography algorithms rely heavily on lithography simulation models. While these algorithms generate high-quality solutions, they suffer from computational complexity and low efficiency. Machine learning, an AI-driven approach, learns patterns from data to make predictions. Techniques such as decision trees and SVMs enable models to classify, regress, and cluster data based on training samples. Machine learning and deep learning have been applied to IC manufacturing, including EUV diffractive imaging for wafer inspection^138^. In recent years, AI has been explored in OPC, SRAF generation, lithography system modeling, and etching effect correction.

Deep learning, a rapidly evolving subset of machine learning, has gained prominence in recent decades^139^. Deep learning extends conventional artificial neural networks by imitating human brain learning mechanisms^140^. Compared to conventional machine learning, deep learning enhances feature learning capabilities through data-driven approaches. Deep neural networks, particularly multi-layer architectures, facilitate deep feature learning and end-to-end optimization. While machine learning relies on manual feature engineering, deep learning autonomously learns data features. In terms of computational complexity, machine learning is suited for small-scale datasets and limited computational resources, and deep learning is appropriate for large-scale data and high-dimensional feature learning. Machine learning is suitable for problems with regularity, while deep learning excels in complex data pattern recognition and high-precision tasks.

**Fig. 7:**
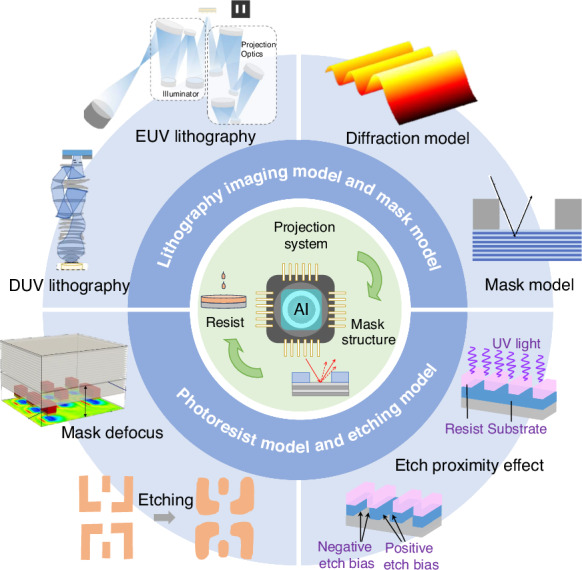
AI for computational lithography modeling, including lithography model, mask model, photoresist model, and etching model. Adapted with permission^51^. Copyright 2013, Society of Photo-Optical Instrumentation Engineers (SPIE). Adapted with permission^59^. Copyright 2019, Optical Society of America. Adapted with permission^60^. Copyright 2022, Optica Publishing Group. Adapted with permission^61^. Copyright 2022, Optica Publishing Group. Adapted with permission^64^. Copyright 2024, Institute of Optics and Electronics, Chinese Academy of Sciences

The advent of deep learning improved AI applications in computational lithography. AI techniques have been employed in various areas, including lithography modeling^141–143^, mask correction^74,144,145^, hotspots detection and correction^75,146–148^, and SRAF generation^149,150^. As shown in Fig. 7, AI techniques contribute to different components of computational lithography, including the lithography model, mask model, photoresist model, and etching model. AI has demonstrated the potential to enhance lithography model accuracy and algorithm efficiency.

For lithography modeling, machine learning aids in feature extraction, error correction, and physical parameter fitting. Techniques such as regression analysis and SVMs enhance lithography models by improving efficiency and interpretability. Machine learning is effective for structured process modeling and local optimization. Deep learning enables high-precision lithography simulations and end-to-end layout optimization. Deep learning is well-suited for generalized learning across multiple process nodes and complex physical modeling. Deep learning offers superior accuracy and adaptability, and also requires significant computational resources and extensive training data. Machine learning is suitable for rapid modeling and error correction, while deep learning excels in high-precision lithography simulation and optimization. The integration of these AI-driven approaches is expected to enhance the efficiency and accuracy of lithography modeling.

### AI-based computational lithography and ILT modeling

#### AI for lithography imaging model and mask model

Fast and accurate lithography models are essential for computational lithography simulations. For lithography systems with NA below 0.6, scalar models effectively describe the imaging process. When NA exceeds 0.6, or even surpasses 1 in ultra-high NA systems, the paraxial approximation in scalar models becomes invalid, and vector models are employed to describe the imaging process^151^. When the exposure wavelength is much longer than the CD of the mask pattern, the Kirchhoff thin mask approximation is adopted^43^. For technology nodes below 45 nm, when the mask CD is comparable to or smaller than the exposure wavelength, a thick mask diffraction near-field (DNF) model is necessary. The mask 3D model accounts for the mask’s structural effects on light amplitude, phase, and polarization. Rigorous electromagnetic field (EMF) simulation can accurately compute DNFs for thick masks. However, the high computational cost makes rigorous EMF simulation impractical for large-area mask simulations and lithography optimization. To address this challenge, fast near-field diffraction calculating methods, including machine learning and deep learning approaches, are widely adopted.

Machine learning techniques help alleviate the computational burden of solving Maxwell’s equations in lithography mask modeling. Ma et al. proposed a machine learning-based method for fast aerial image calculation of thick masks in partially coherent lithography systems^49^. By using sparse sampling points and training libraries of thick-mask DNFs, the method enhanced computational efficiency and accuracy, with validations at the 45 nm and 14 nm nodes. Lin et al. introduced a machine learning approach for 3D mask near-field calculations^50^. The approach synthesized complete mask near fields using non-parametric regression, data fusion, and image stitching. The method outperformed the rigorous EMF simulators. Liu et al. extended a fast approximate 3D mask model, originally designed for deep ultraviolet (DUV) applications^51^. The enhancement improved the prediction and correction of 3D mask topography effects. Zhang et al. proposed a machine learning-based parameter correction method for simulating 3D defect masks in EUV lithography^52^. The approach improved both simulation accuracy and computational speed. As indicated by advancements in fast aerial image calculations, 3D mask near-field modeling, and defect simulation, machine learning techniques demonstrate the potential to enhance computational efficiency and accuracy for lithography mask modeling.

Deep learning is also applied to mask modeling. Tanabe et al. explored the limitations of the thin mask model in EUV lithography and proposed using a CNN to accelerate calculations^53^. They also extended the transmission cross coefficient formula for off-axis mask 3D effects. Lin et al. developed a fully convolutional network (FCN) method to calculate thick mask near-fields^54^. The model was trained on rigorous EMF simulation data. The method improved efficiency in EUV lithography simulations compared to rigorous EMF methods.

Deep learning has further advanced computational lithography modeling. Kareem et al. proposed a method for synthesizing lithography test patterns using image parameter space values. The approach, based on an adversarial auto-encoder and an auto-encoder, improved pattern diversity and simulation accuracy^55^. Ye et al. introduced LithoGAN, a GAN-based lithography modeling framework. LithoGAN directly mapped mask patterns to resist patterns with high accuracy while reducing computational costs^56^. Ye et al. presented TEMPO, a generative learning-based framework for 3D aerial image prediction. TEMPO predicted aerial image intensity for different resist heights, achieving a speedup of up to 1170 times compared to rigorous simulation^57^.

Machine learning and deep learning techniques enhance lithography mask modeling by improving prediction accuracy and computational efficiency. By integrating machine learning with rigorous EMF simulations, the methods enhance performance in handling complex masks and 3D effects. Research in thick mask modeling and near-field calculations demonstrates the applicability of machine learning across different technology nodes. Deep learning expands the possibilities. CNNs and GANs are used to reduce computational costs and increase pattern diversity and accuracy.

**Fig. 8:**
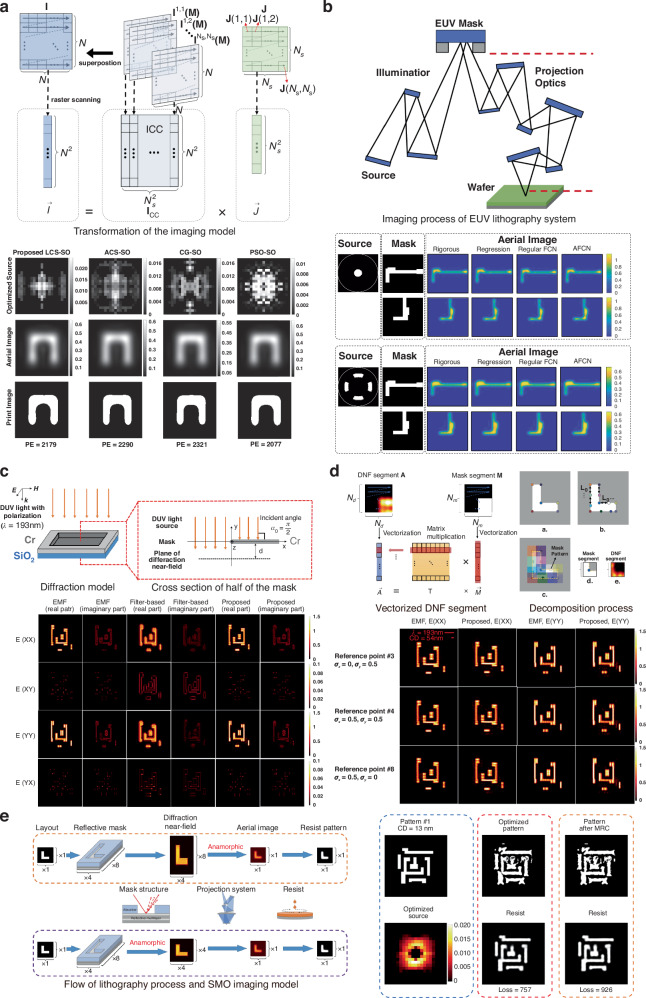
AI-based methods for lithography optimization. **a** Learning-based source optimization method under the compressive sensing framework for EUV lithography. The transformation of the imaging model demonstrates the correlation between source pattern and aerial image. The simulation results obtained by the learning-based source optimization method are shown. Reproduced with permission^59^. Copyright 2019, Optical Society of America. **b** EUV lithography aerial image model based on an AFCN for thick-mask effects and oblique incidence. The aerial images of test masks generated by conventional and quasar sources are presented. Adapted with permission^60^. Copyright 2022, Optica Publishing Group. **c** Thick-mask model based on the DTM for thick-mask diffraction simulation by learning local DNF characteristics. The diffraction model, the real part and the imaginary components of the diffraction matrices are shown. Reproduced with permission^61^. Copyright 2022, Optica Publishing Group. **d** Learning-based thick-mask model for thick-mask DNF simulation in immersion lithography. The figure demonstrates the relationship between vectorized DNF and mask, the decomposition process, and the amplitudes of diffraction matrices under oblique illumination. Adapted with permission^62^. Copyright 2023, Optica Publishing Group. **e** Source-mask co-optimization method for high-NA EUV lithography. The SMO process flow, the target patterns and the optimized masks are shown. Adapted with permission^64^. Copyright 2024, Institute of Optics and Electronics, Chinese Academy of Sciences

Recent research demonstrated the transformative role of AI in lithography optimization and simulations. Lan et al. proposed a deep neural network (DNN)-based GPU-accelerated mask optimization platform. The approach reduced runtime without compromising accuracy, addressing challenges in RET for sub-10 nm curvilinear masks^58^. Lin et al. developed a fully convolutional network for thick mask near-field calculations^54^. A source optimization method based on compressive sensing was introduced^59^. Sparse sampling and joint optimization of the source dictionary and projection matrix were adopted. The method improved imaging fidelity and computational efficiency. The transformation of the imaging model and the source optimization results are shown in Fig. 8a. A fast EUV lithography aerial image model based on an adjoint fully convolutional network (AFCN) was investigated^60^. A dual-path architecture was employed to separately recover the real and imaginary components of the mask DNF. Feature-swapping techniques were incorporated to enhance accuracy. The aerial images of testing masks are shown in Fig. 8b.

Li et al. developed several techniques for thick-mask modeling and 3D photomask simulation. The diffraction transfer matrix (DTM) approach improved accuracy and efficiency in thick-mask simulations^61^. They extended models to PCI at 14 nm nodes^62^, and proposed a fast asymmetric patch data fitting method^63^. An improved thick-mask model was proposed using DTM to simulate 3D mask diffraction in optical lithography systems^61^. The DTM, trained on rigorous DNF data, enabled efficient synthesis of overall DNFs by decomposing mask patterns and integrating local diffraction effects. The diffraction model and the diffraction matrices are shown in Fig. 8c. A learning-based thick-mask model was developed to achieve high-precision DNF simulations under PCI conditions^62^. A rigorously trained oblique illumination DNF library was leveraged. Computational efficiency was improved by up to two orders of magnitude compared to the EMF simulator. The relationship between vectorized DNF and mask, the decomposition process, and the amplitudes of diffraction matrices under oblique illuminations are shown in Fig. 8d. An SMO method was proposed to reduce pattern errors in high-NA EUV lithography^64^. A high-NA EUV lithography model was established, with gradient-based mask optimization and compressive sensing-based source optimization. The optimized mask pattern was simplified through the mask rule check (MRC) process. The SMO process, the target patterns and optimized masks are shown in Fig. 8e. Machine learning and deep learning revolutionize lithography modeling by improving accuracy, efficiency, and process control. Neural networks effectively address challenges in thick-mask modeling, near-field calculations, and source optimization. AI approaches improve the lithography process control, becoming valuable complements or alternatives to conventional simulation methods.

#### AI for photoresist model and etching model

Lithography resist effect can be described by rigorous physical models or simplified compact models. Rigorous models employ PDEs to describe the physical and chemical changes during exposure and development. However, rigorous models suffer from computational complexity and inefficiency. Simplified compact models, such as the variable threshold resist model and the hard threshold model, offer a practical alternative. Etch bias is a crucial metric in computational lithography. Etching process models are categorized into physical models and empirical models. Physical models provide an accurate description of the etching profile, but require a deep understanding of the underlying physical principles. Empirical models, also known as black-box models, bypass the fundamental physics and instead use input-output data relationships. Common fitting methods include least squares, principal component regression, and neural networks. Although empirical models rely on experimental data and lack physical interpretability, they are widely used in manufacturing due to their practicality.

As the CDs of ICs shrink, etching deviation tolerance requirements become increasingly stringent. Researchers have turned to machine learning and deep learning techniques for photoresist and etching simulations. Xia et al. employed a neural network to analyze electron cyclotron resonance etching, identifying optimized etch rate peaks influenced by process parameters and providing detailed physical interpretations of the etching process^65^. Kim et al. developed a neural network optimized with a genetic algorithm for silicon oxynitride etching, achieving 52% improvement over regression models and providing insights into the etch mechanisms^66^. Liu et al. extended a fast approximate 3D mask model for DUV lithography to predict and correct 3D mask topography effects in EUV applications. Shiraishi et al. improved flare calculation by introducing two point spread functions to account for scattered light reaching the wafer, considering energy loss of optics^67^. Shim et al. applied machine learning techniques, such as SVM and neural networks, to lithography tasks, including OPC, SRAF insertion, and etch proximity correction (EPC)^68^. Shim et al. further introduced an artificial neural network (ANN)-based EPC method that predicts etch bias using geometric and optical parameters, improving pattern fidelity in 20 nm DRAM gate layers^69^. Chen et al. presented a machine learning-based etch bias prediction model for both one-dimensional (1D) and 2D layouts, achieving absolute errors below 2 nm for 1D patterns and below 4 nm for 2D layouts, demonstrating its potential for high-volume manufacturing of model-based EPC^70^. Pan et al. developed an informatics-based SMO method, employing a communication channel model and mutual information optimization to improve imaging performance and reduce patterning errors^71^. The studies demonstrate how machine learning and deep learning enhance accuracy and efficiency in etching, photoresist simulation, and lithography optimization.

Machine learning and deep learning techniques are successfully explored and applied in the lithography and etching processes. Neural networks and SVMs have improved performance in etch profile variation, etch rate optimization, and photoresist modeling. Some approaches integrate genetic algorithms with physical models to enhance interpretability and accuracy in etching simulations. The methods accelerate computation, reduce design errors, and promote feasibility for high-volume manufacturing. By addressing complex, multi-variable challenges in photoresist and etching processes, AI enhances robustness and efficiency in semiconductor manufacturing.

### AI-based computational lithography and ILT acceleration algorithm

Model-based computational lithography algorithms rely on iteratively employing lithography models to generate high-quality solutions. But model-based methods are faced with challenges of computational complexity and low efficiency. Compared to RBOPC and EBOPC, PBOPC performs pixel-level corrections on masks, offering greater flexibility and computational speed. The introduction of machine learning and deep learning provides new avenues for fast lithography simulations. Network training replaces the complex iterative process, improving the efficiency of the algorithm.

Researchers have employed machine learning to accelerate the PBOPC, including adaptive kernel regression^72,152^, SVM^73^, and multilayer perceptron (MLP)^74^. The algorithms improve computational efficiency and hold the potential for higher simulation accuracy and imaging quality. Ma et al. proposed a machine learning-based OPC algorithm to reduce the computational intensity and mask complexity, achieving two-fold speedup and more manufacture-friendly OPC patterns compared to professional PBOPC software^152^. Ma et al. presented a PBOPC algorithm using nonparametric kernel regression, leveraging machine learning to accelerate the computation and reduce mask complexity^72^. Luo et al. proposed an SVM-based layout retargeting method for ILT, reducing the optimization iterations and runtime by 70.8% and 69.0%, respectively, by generating an initial input mask close to the final optimized mask^73^. Luo et al. utilized a multilayer perceptron neural network trained via backpropagation to generate OPC mask patterns, achieving high pattern fidelity without iteration^74^.

**Fig. 9:**
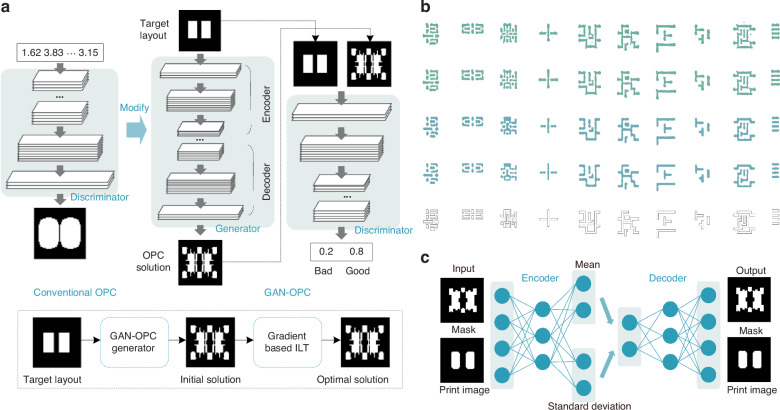
AI implementations for computational lithography and inverse lithography algorithms. **a** Inverse lithography-guided GAN-OPC for mask optimization^76^. Based on conventional GAN, the GAN-OPC improves by employing an auto-encoder structure composed of an encoder and a decoder. **b** Optimization results of PGAN-OPC and ILT, with each column representing a test pattern. From top to bottom, the rows display optimized masks of ILT, optimized masks of PGAN-OPC, wafer patterns of ILT, wafer patterns of PGAN-OPC, and target patterns. Reproduced with permission^76^. Copyright 2018, Association for Computing Machinery (ACM). **c** VAE-based inverse layout design method^79^

Deep learning demonstrates advantages in data processing, feature extraction, and nonlinear prediction, showing superior computational speed^153^. Various deep learning models have been applied in computational lithography, including CNNs^54,75^, GANs^76^, variational autoencoders (VAEs)^79^, and graph convolutional neural networks (GCNs)^80^. Yang et al. proposed a GAN-based mask optimization method to address the high computational demands of advanced lithography technology nodes^76^. By developing an OPC-oriented GAN flow, the method learns target-mask mapping and incorporates ILT for pre-training. Improved upon conventional GAN, the model employs an auto-encoder structure composed of an encoder and a decoder, as illustrated in Fig. 9a. The GAN-OPC flow learns nonlinear target-mask mapping and produces optimized masks. The ILT-guided pre-training approach improves the training speed and predictive capability of the generator. The experiment records the training behaviors of GAN-OPC and PGAN-OPC. PGAN-OPC denotes the GAN-OPC flow with ILT-guided pre-training. Figure 9b illustrates the optimization results of the PGAN-OPC and ILT, with each column representing a test pattern. From top to bottom, each row displays: optimized masks of ILT, optimized masks of PGAN-OPC, wafer patterns of ILT, wafer patterns of PGAN-OPC, and target patterns. The L_2_ denotes the squared L_2_ error between the wafer image and the target image under nominal conditions. The average L_2_ of ILT is 44,012.7, the average L_2_ of GAN-OPC is 40,094.6, and the average L_2_ of PGAN-OPC is 39,948.9. Both GAN-OPC and PGAN-OPC reduce the squared L_2_ error of the wafer image by 9%. The PVB denotes the contour area process variation band under ±2% dose error. The average PVB of ILT is 50,899.5, the average PVB of GAN-OPC is 50,568.1, and the average PVB of PGAN-OPC is 49,957.2. The average runtime of ILT, GAN-OPC, and PGAN-OPC is 788.5 s, 384.7, and 371.3 s, respectively. GAN-OPC and PGAN-OPC achieve an average reduction of 50% in runtime compared to ILT. Experimental results demonstrate that the method accelerates computation and enhances mask fidelity.

The application of deep learning in computational lithography covers various research such as hotspot detection, mask optimization, and layout design. Yang et al. presented a deep CNN for lithography hotspot detection focusing on representative feature learning^75^. The imbalance problem in the training dataset is tackled through hotspot up-sampling techniques. Sim et al. proposed a deep generative network for lithography hotspot correction, employing an optimized learning strategy^77^. Derived from GAN, CycleGAN is a deep generative model for unpaired image-to-image translation between different domains^78^. The key idea of cycleGAN is to couple the training of forward and inverse mappings, and use a cycle consistency loss to guarantee the relationship. The lithography hotspot correction can be considered as translating hotspots into cold-spots. Zhang et al. proposed a VAE-based design method to address layout designs with complex constraints^79^, as shown in Fig. 9c. The VAE’s learning and generative capabilities were utilized to automatically satisfy constraints. The approach demonstrated efficiency in inverse design for surface diffusion and mask design in optical microlithography. Zhang et al. presented a GCN-based pixelated OPC method to improve computational efficiency in the lithography process^80^. The GCN was utilized to predict OPC solutions pixel by pixel from raster-scanned target layouts. Simulation results validated the method’s efficiency compared to traditional methods. The studies showcase the advancements of deep learning in computational lithography, and provide efficient solutions to issues in semiconductor manufacturing.

Standard deep learning techniques face several shortcomings, including complex network design, limited physical interpretability, and unpredictable performance. A potential solution is incorporating prior physical models to assist in DNN design and training. Researchers have proposed model-driven deep learning (MDL) methods^81–84^. MDL is a paradigm that integrates physical models with data-driven approaches. By incorporating domain knowledge, such as physical laws and mathematical models, MDL constructs models that are physically consistent and interpretable. The core idea of MDL is to leverage physical models to provide structured constraints and prior knowledge, while optimizing parameters with data. MDL approaches improve model generalization, interpretability, and accuracy. Unlike conventional deep learning, which relies purely on data-driven approaches, MDL explicitly integrates physical models and domain expertise. Conventional deep learning methods are considered as black boxes, lacking transparency. MDL enhances interpretability, physical consistency, and generalization by introducing physical constraints to ensure the model’s outputs align with physical laws. The advantages of MDL include reduced reliance on large-scale labeled data, improved data efficiency, and enhanced model robustness.

**Fig. 10:**
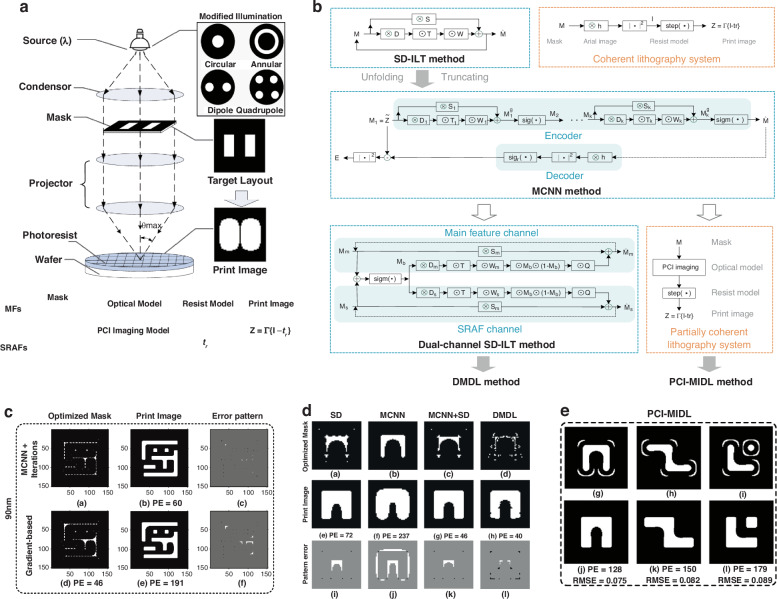
Model-driven deep learning methods for lithography optimization. **a** Physical and mathematical representation of the lithography system. Light from the source passes through the mask, forming an image on the wafer surface. After photoresist development and etching, the printed pattern emerges. Adapted with permission^84^. Copyright 2020, Optical Society of America. **b** Structure of MDL-based computational lithography and ILT methods. For coherent lithography, the MCNN framework is designed by unfolding and truncating the gradient-based ILT (SD-ILT) algorithm. The DMDL introduces parallel channels to simultaneously predict MFs and SRAFs. The PCI-MIDL employs a dual-channel architecture to accommodate the lithography system with PCI. **c**–**e** demonstrate the optimized masks, printed images, and pattern errors of MCNN^81^, DMDL^84^ and PCI-MIDL^83^ approaches. Adapted with permission^81^. Copyright 2018, Optical Society of America. Reproduced with permission^84^. Copyright 2020, Optical Society of America. Adapted with permission^83^. Copyright 2020, Optical Society of America

MDL utilizes physical models as prior information. Some parameters remain undetermined, forming a model family. Based on the model family, objective functions and iterative algorithms are constructed to form an algorithm family. The iterative algorithms are unfolded and truncated to complete the initial design of the network structure and parameter initialization. Researchers have applied MDL to lithography simulation, proposing a series of MDL-based fast computational lithography techniques, as presented in Fig. 10. Ma et al. proposed an MCNN framework to approximate ILT solutions, reducing computational complexity and improving lithography image fidelity in coherent optical systems^81^. The MCNN network structure is constructed by unfolding and truncating the gradient-based ILT algorithm^82^. The lithography imaging model serves as a decoder for network training. Ma et al. presented a DMDL method to improve image fidelity and computational efficiency in ILT^84^. The DMDL divides data flow into parallel channels, enabling simultaneous prediction of main features (MFs) and SRAFs. Zheng et al. proposed a model-informed deep learning (MIDL) approach to improve the computational efficiency and image fidelity of optical lithography systems with PCI^83^. The PCI-MIDL has a dual-channel feature, predicting the MFs and SRAFs simultaneously. The Fourier series expansion imaging model is used as the decoder for training. MDL enhances interpretability and accuracy by integrating lithography imaging models. MDL adjusts model parameters through a data-driven approach, improving image fidelity. MDL reduces computational complexity and maintains physical consistency, making the model interpretable and reliable.

## Challenges and strategies for inverse lithography technology

**Fig. 11:**
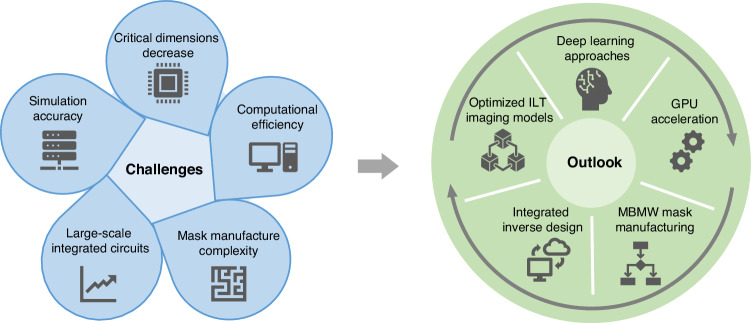
**Challenges and outlook of ILT**. The challenges include the critical dimensions decrease, simulation accuracy, computational efficiency, large-scale integrated circuits, and mask manufacturing complexity. The outlook includes using deep learning approaches, developing optimized ILT imaging models, employing GPU acceleration, utilizing integrated inverse design, and investigating advanced mask manufacturing approaches such as MBMW

The semiconductor industry continually seeks faster simulation technologies to accelerate the advancement of the next-generation lithography process. At technology nodes of 7 nm and below, the increased device integration and shrinking CDs have significantly raised data processing loads and computational complexity. The current challenges of ILT are shown in Fig. 11. Computational requirements pose substantial challenges to simulation efficiency. Various optical effects, system errors, and process deviations significantly impact the lithography imaging quality. To enhance the yield of chip manufacturing, it is essential to improve simulation accuracy and imaging fidelity. ILT is expected to integrate with advanced mathematical and signal processing methods to achieve advancements in model frameworks, simulation principles, and algorithm performance.

### Optimization efficiency

An important challenge faced by ILT is the optimization efficiency. ILT and OPC share similarities in optical and resist process modeling. However, ILT and OPC models differ in several aspects. ILT is an optimization process that relies on cost function derivatives. Curvilinear ILT models should account for all angles rather than just horizontal and vertical edges, so conventional fast-computing techniques for Manhattan patterns are unsuitable. The traditional model-based computational lithography relies on iterative simulations. Though generating high-quality solutions, the methods suffer from high computational costs and inefficiency. With the same computational resources, ILT requires runtime more than an order of magnitude longer than OPC. Due to the runtime constraints, ILT is primarily used for hotspot corrections. To manage computational limitations, full-chip ILT is divided into multiple layout units, which are optimized separately and then stitched together. The strategy results in discrepancies in ILT-optimized mask patterns, particularly SRAFs at unit boundaries, leading to stitching issues and inconsistencies. Moreover, the curvilinear ILT patterns should undergo Manhattanization for manufacturable mask production. The process of Manhattanization, layout optimization, and stitching increases the overall runtime.

Machine learning and deep learning have found widespread applications in computational lithography fields such as OPC, SMO, SRAF generation, lithography system modeling, and etching effect correction. AI has shown promise in improving model and algorithm efficiency. However, existing deep learning technologies have inherent shortcomings, including difficulty in network design, poor physical interpretability, and unpredictable performance. Combining computational lithography principles with advanced data processing technologies is a promising direction. Machine learning and deep learning heavily rely on training samples, but the cost of generating and collecting large amounts of data is huge. Deep learning methods depend on black-box models for prediction, which lack physical interpretability, making issue analysis and correction difficult. Current deep learning models struggle to precisely optimize lithography, often yielding approximate rather than exact solutions.

In the foreseeable future, computational lithography and ILT will continue to grapple with constraints on computing speed and optimization efficiency. Achieving fast and accurate computational lithography for large-scale IC layouts is a crucial goal. With advancements in theories and algorithms, AI is expected to bring a new leap forward in computational lithography technology. Ensuring the stability and reliability of AI in lithography applications is a critical avenue for exploration.

### Mask manufacturability

ILT mask fabrication faces several challenges, including the immaturity of mask process correction (MPC) and curvilinear mask data processing techniques, as well as the long time required for VSB mask writing. Rather than limited to right angles or straight lines, ILT-generated curvilinear mask features curved geometric shapes. By optimizing pattern edge curvature, curvilinear masks reduce OPEs and enhance imaging resolution of wafer patterns. The advantages of curvilinear masks over rectilinear designs include the reduction of optical diffraction effects, improvement of OPEs, enhancement of resolution and pattern transfer accuracy, increased design flexibility, and optimization of the process window. Curvilinear masks are typically applied in advanced nodes, such as 7 nm, 5 nm, and beyond, to meet the demanding requirements for higher resolution and precision in lithography process.

During mask fabrication, the pattern resolution is influenced by factors such as electron beam dose distribution and energy contrast, resist resolution, and etching processes. MRC is a necessary step before mask writing to ensure compliance with manufacturing rules and to optimize any non-compliant regions. For Manhattanized ILT mask patterns, MRC rules include minimum line width, spacing, area, and diagonal corner spacing. For curvilinear masks, additional MRC involves minimum curvature in concave and convex features. Integrating MRC into full-chip ILT ensures that optimized curvilinear mask patterns remain manufacturable.

The mask fabrication process includes two main steps, mask data processing and mask manufacturing. In EB mask writing process, EB proximity effects require corrections to OPC-processed layouts, referred to as MPC. Mask data preparation (MDP) converts the layout into a format compatible with EB machines, segmenting the complete layout into EB shots. After the process of ILT, MRC, MPC, and MDP, the mask layout becomes ready for manufacturing.

EB lithography is crucial for advanced IC production. Modern mask fabrication relies on two primary EB writing techniques: single variable-shaped beam (SVSB) writing and MB writing^154^. SVSB writing has been predominantly employed for nodes down to 5 nm, while MB writing is utilized for nodes beyond this threshold^155^. SVSB divides complex patterns into variable-shaped shots to reduce the shot count. This efficiency makes SVSB systems integral to mask manufacturing. However, as ILT introduces intricate curvilinear designs, SVSB struggles to efficiently write such patterns. Writing complex curve patterns by SVSB requires excessive shot division and prolongs writing times. To address this problem, MB mask writing systems are developed. In MB mask writing system, mask data is rasterized into pixel data. The writing time remains constant regardless of pattern size or complexity, as the pixel area remains uniform. The characteristics make MB mask writing systems essential for mask production. Writing curvilinear ILT masks using variable-shaped beams (VSB) is challenging and time-consuming. The curvilinear mask shapes provide large process windows and exhibit superior manufacturing resilience^156,157^. Researchers are actively investigating solutions to design curvilinear mask-writing tools.

Balancing mask complexity with writing efficiency is critical. ILT masks with SRAFs demonstrate superior process window. However, SRAFs also extend the mask writing time. One strategy is to restrict the regions of SRAFs^158,159^. At advanced nodes, the sharp corners in the Manhattan mask shapes tend to become more rounded during resist process and absorber etch. It is possible to approximate an ideal curved mask with relatively coarse Manhattan segments.

Despite advancements, full-chip curvilinear ILT mask patterns remain challenging for VSB writers^160^. Studies of ASML Biron and Numerical Control System indicated that while VSB could write stair-cased ILT patterns, the time is prohibitively lengthy^161^. Mask rules for curvilinear ILT remain an area of active research. Design-to-Silicon proposed an implementation of mask rules based on the concept of two circles^157^. The curvature is checked by sliding circles along the pattern edge. If any overlap occurs between the circle and the pattern contour, the curvature is deemed unreliable for manufacturing.

MB mask writing is evolving to meet high-volume manufacturing demands. The MB writing is developed with specialized features tailored for high-volume production. The features include high beam current density, a single-stage acceleration optical beam blanking aperture array system, rapid inline pixel-level dose correction, and hardware for minimizing charging effects^154^. The mask writing techniques together enhance the performance of the mask writing process.

### Outlook

Future research will focus on refining ILT algorithms, optimizing deep learning approaches for lithography, and developing advanced mask manufacturing techniques, as shown in Fig. 11. Integrated designs of forward imaging simulation models and inverse optimization algorithms offer promising avenues for future research of ILT. Leveraging advanced computer software and hardware to construct high-speed platforms helps to alleviate the computational burden.

Integration of ILT with AI offers promising opportunities to improve lithographic performance and mask manufacturability. AI can aid ILT by generating models for optical systems, 3D mask structures, and photoresist behavior. Leveraging deep learning and reinforcement learning, a fully automated mask pattern generation workflow could achieve results comparable to ILT optimization. In 2023, NVIDIA showcased its ILT product, CuLitho, at the developer conference, achieving a 40-fold increase in ILT computation speed on a GPU platform. As the semiconductor industry demands greater computational power, GPU-accelerated deep learning is expected to become the mainstream.

As semiconductor manufacturing advances, lithography technology faces increasingly complex challenges. Conventional data-driven deep-learning approaches are limited by data scarcity and a lack of physical consistency. MDL integrates physical models with deep learning, providing new directions for future research. Studies on MDL in computational lithography and ILT focus on enhancing physical consistency, interpretability, and computational efficiency. An area of exploration is the integration of multi-scale physical models within the MDL framework. In lithography imaging, physical effects such as optical diffraction and photoresist reactions can be incorporated into deep learning frameworks through hierarchical modeling. In source and mask optimization, MDL can introduce physical constraints to ensure solutions align with manufacturing requirements. The interpretability and transparency of MDL enhance the reliability for industrial applications. By incorporating physical models within network architecture, MDL generates physically consistent results. As a paradigm that combines physical modeling with data-driven techniques, MDL is expected to play a crucial role in computational lithography and ILT.

The use of advanced mask fabrication tools, such as multi-beam mask writers (MBMW), is expected to address the challenges associated with ILT mask production, including large number of exposure units, long writing times, and high costs. MBMW systems can achieve sub-10 nm writing resolution while maintaining efficiency. By increasing the edge dose, the edge slope is enhanced, and small feature resolution is enhanced. Direct MBMW writing of ILT masks is a viable solution. By addressing these challenges, the semiconductor industry can fully harness the potential of ILT, paving the way for the development of next-generation very large scale integration circuit (VLSI).

## Conclusion

In this review, we introduce the principles and development of ILT, with a focus on AI integration within ILT frameworks. Recent research has advanced computational lithography models and optimization algorithms. AI techniques, including machine learning and deep learning, have significantly contributed to lithography models, including imaging models, mask models, photoresist models, and etching models. Additionally, AI has been applied to accelerate computational lithography algorithms, employing methods such as GANs, CNNs, VAEs, and MDLs.

Despite progress, several obstacles limit the widespread application of ILT in semiconductor manufacturing. As ICs become complex and CDs decrease, primary challenges include simulation accuracy, computational efficiency, runtime, mask manufacturing complexity, and mask writing time. Addressing these obstacles is crucial for ILT’s integration into high-volume production. The development of computational lithography and ILT requires a balanced consideration of multiple factors, including computational accuracy, efficiency, optimization freedom, and mask manufacturability, which often conflict. Integrating forward imaging simulations with inverse optimization is a research trend. Leveraging advanced software and hardware for high-speed platforms helps to reduce ILT’s computational burden. GPU-based deep learning also holds promise for improving lithography optimization. Future research will refine GPU-accelerated deep learning-based ILT algorithms, and develop advanced mask manufacturing techniques, unlocking ILT’s full potential for next-generation VLSI designs.

## Data Availability

The data supporting this research is available from the corresponding authors upon request.
